# The BIOPREVENT machine-learning algorithm predicts chronic graft-versus-host disease and mortality risk using posttransplant biomarkers

**DOI:** 10.1172/JCI195228

**Published:** 2026-02-16

**Authors:** Michael J. Martens, Debjani Dutta, Yongzi Yu, Lisa E. Rein, Jerome Ritz, Brent R. Logan, Sophie Paczesny

**Affiliations:** 1 Division of Biostatistics and; 2 Center for International Blood and Marrow Transplant Research, Medical College of Wisconsin, Milwaukee, Wisconsin, USA.; 3 Hollings Cancer Center and; 4 Department of Pharmacology and Immunology, Medical University of South Carolina, Charleston, South Carolina, USA.; 5 Department of Medical Oncology, Dana-Farber Cancer Institute and Harvard Medical School, Boston, Massachusetts, USA.

**Keywords:** Clinical Research, Hematology, Biomarkers, Machine learning, Proteomics

## Abstract

**BACKGROUND:**

Chronic graft-versus-host disease (cGVHD) is a major contributor to nonrelapse mortality (NRM) following hematopoietic cell transplantation (HCT). Whether machine-learning (ML) models with biomarkers improve the accuracy for predicting future cGVHD/NRM is not established.

**METHODS:**

We developed BIOPREVENT (BIOmarkers PREVENTion), a ML algorithm using data from 1,310 HCT recipients, incorporating 7 plasma proteins measured at Day 90/100 post-HCT and 9 clinical variables. Patients were divided into training and validation datasets. ML models — including CoxXGBoost, Group SCAD, Adaptive Group Lasso, Random Survival Forests, and Bayesian Additive Regression Trees (BART) — were used to estimate time-varying Area Under the ROC Curve (AUCt) at Days 180, 270, 360, and 540. Deep learning models were also evaluated.

**RESULTS:**

ML models with biomarkers outperformed clinical-only models for predicting cGVHD, with BART and CoxXGBoost achieving AUCt greater than 0.65 at 1 year. For NRM, models with biomarkers achieved AUCt ranging from 0.75–0.91. Deep learning did not outperform other ML approaches. BART consistently demonstrated high predictive accuracy and was selected for the final BIOPREVENT model. Calibration curves aligned with observed values. Variable importance analysis identified MMP3 and CXCL9 as key for cGVHD prediction and IL1RL1 and sCD163 for NRM. Cumulative incidences of cGVHD and NRM differed significantly based on BIOPREVENT-defined cutpoints.

**CONCLUSION:**

BIOPREVENT accurately predicts individual risk of future cGVHD and NRM using biomarkers at 3 months post-HCT. A publicly available R Shiny web application supports its clinical use. Further studies are needed to explore its role in guiding preemptive therapy.

**TRIAL REGISTRATION:**

BMTCTN 0201, BMTCTN 1202, and NCT02194439.

**FUNDING:**

R01CA264921, U10HL069294, U24HL138660, R01HD074587, and P01HL158505.

## Abstract 2

BIOPREVENT, a machine learning (ML)-derived web application, can accurately predict an individual’s future cGVHD and death using biomarkers post-transplant to enable personalized screening.

## Graphical abstract

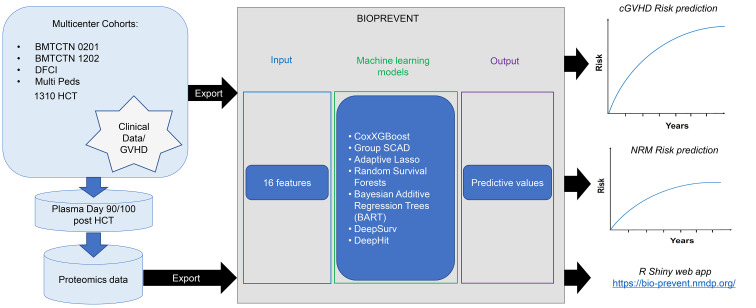

## Introduction

Allogeneic hematopoietic cell transplantation (HCT) is a potentially curative therapy for blood diseases. However, its efficacy has been impeded by chronic graft-versus-host disease (cGVHD), a leading cause of nonrelapse mortality (NRM) and debilitating morbidity (1, 2). Transplant approaches such as cord blood transplantation, T-cell depletion, antithymocyte globulin (ATG)/alemtuzumab, and posttransplant cyclophosphamide (PTCY) and better supportive care have decreased the overall incidence of cGVHD (3). However, needs and opportunities to refine preemptive therapy approaches have also been recently highlighted (4).

The diagnosis of cGVHD continues to be assessed by nonspecific clinical symptoms in many organs (5) and represents the culmination of tissue perturbations initiated by donor cell infusion. The NIH cGVHD consensus determined that identifying risk biomarkers was a priority to enable preemptive treatment (1, 2, 6, 7). Our previous work identified and validated risk biomarkers for the future development of cGVHD in individuals without clinically apparent disease (8). Biomarker levels of C-X-C chemokine ligand 9 (CXCL9), matrix-metalloproteinase-3 (MMP3), and dickkopf-WNT signaling pathway inhibitor-3 (DKK3) measured at 3 months post-HCT identify patients at risk for cGVHD occurrence. An algorithm including CXCL9+MMP3+DKK3 created a score with specificity over 50% and 75% sensitivity (8). Furthermore, patients with 1 log_n_ increase in IL-1 receptor like 1 (IL1RL1, formerly ST2) had 1.6–2.0 times higher NRM (8). Graft source (peripheral blood stem cells [PB] versus bone marrow [BM]) was also identified as an important independent clinical risk factor for developing cGVHD (8). However, whether machine learning (ML) algorithms could improve prediction of cGVHD and NRM is unknown.

This study aimed to enhance risk stratification for cGVHD and NRM by applying advanced ML techniques to clinical and biomarker data measured at Day 90/100 post-HCT. Specifically, we analyzed 7 previously validated plasma biomarkers alongside key clinical variables to develop predictive models of these 2 posttransplant outcomes. The dataset comprised 1,310 HCT recipients drawn from 4 well-characterized, multicenter cohorts: BMTCTN 0201 and 1202, the Dana-Farber Cancer Institute (DFCI), and a combined pediatric/adult cohort from trial NCT02194439. In addition to building robust ML-based prediction models, the study explored 2 secondary aims: (a) whether deep learning approaches, also called artificial intelligence, offer superior predictive performance compared to traditional ML algorithms, and (b) the feasibility of translating these models into a user-friendly, web-based clinical decision tool.

## Results

### Patient demographics, cGVHD and NRM.

**Figure 1:**
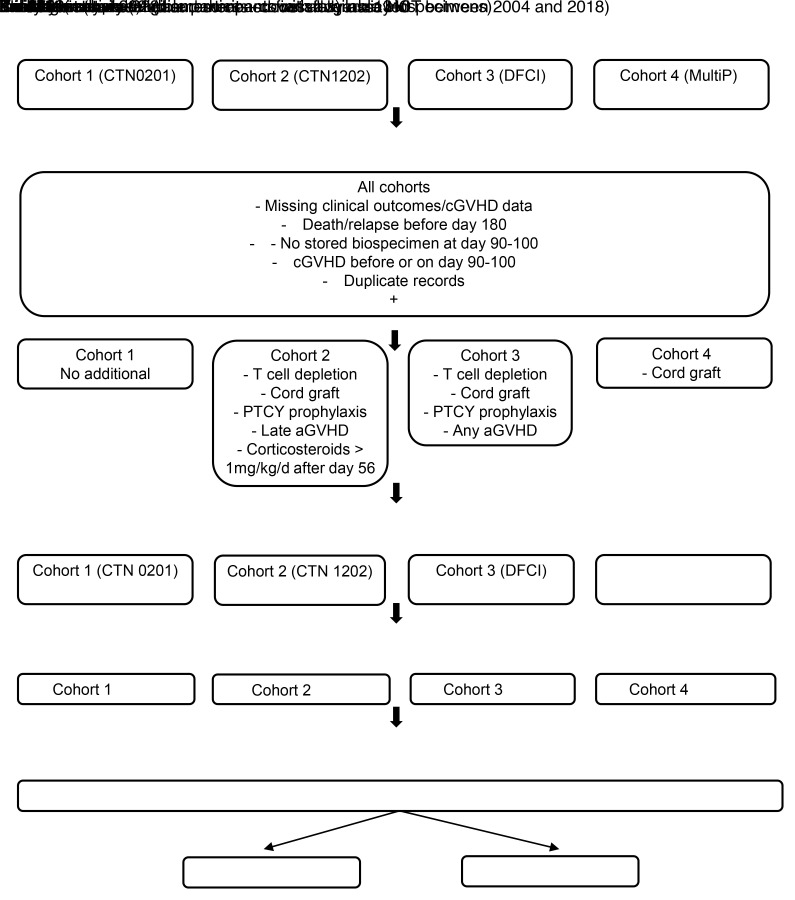
CONSORT Flow Diagram. This diagram summarizes participant inclusion across 4 retrospective cohorts. Individuals were screened for eligibility based on available clinical data and availability of biospecimens. The number of participants excluded at each step is indicated along with reasons for exclusion (e.g., missing clinical data, cGVHD before or on day 90/100, or late aGVHD). The final analytic dataset comprises 4 cohorts with total participant numbers shown for each cohort. The diagram outlines all steps from initial identification through exclusions and biomarker measurements.

The CONSORT diagram for this analysis shows that the primary analysis set (biomarkers and covariates) included 1,310 patients with HCT with a sample available at day 90/100 after HCT. This set was randomly split into training and validation sets comprising 75% and 25% of the patients, respectively (Figure 1). Characteristics of the 1,310 patients with HCT are shown in Table 1, stratified by cohort and analysis set (training versus validation). There was greater representation of nonmalignant diseases, lymphoblastic malignant disease, BM grafts, and GVHD prophylaxis with ATG/PTCY among younger recipients. The proportions of patients having any cGVHD was 52%, moderate to severe cGVHD was 37%, and NRM was 15%. Event frequencies were well balanced across training and validation sets (Supplemental Table 1; supplemental material available online with this article; https://doi.org/10.1172/JCI195228DS1).

### Univariate assessments of biomarkers for cGVHD risk.

All 7 biomarkers were measured in accordance with the manufacturers’ protocols (Supplemental Table 2). They were then assessed for association with cGVHD occurrence in univariate Cox models. Biomarker levels from the full cohort are shown in Supplemental Table 3. Moderate Spearman correlations of 0.28, 0.35, and 0.35 were observed between the respective pairs CXCL9, CXCL10; CXCL10, IL17; and CXCL9, sCD163; and 0.45 and 0.54 between the respective pairs MMP3, DKK3 and MMP3, IL1RL1 (Supplemental Figure 1). Cox models of cGVHD showed all markers except sCD163 were correlated with cGVHD (Supplemental Table 4).

### Multivariate ML models of cGVHD risk.

Using ML techniques, the biomarkers and 9 clinical variables were assessed for their association with cGVHD occurrence. ML methods considered included Cox regression with Group Smoothly Clipped Absolute Deviation (SCAD) (9) and Adaptive Group Lasso (10) penalties, Cox regression with extreme gradient boosting (CoxXGBoost) (11), Random Competing Risks Forests (12), Oblique Random Survival Forests (13), Bayesian Additive Regression Trees (BART) (14), and deep learning via DeepSurv (15) and DeepHit (16). Deep learning constitutes the subset of ML models that employ deep neural networks. All models of cGVHD, moderate/severe cGVHD, NRM, and relapse were landmark analyses at Day 90/100 including patients still at risk for the event. Each ML model was constructed using the training set; its predictive accuracy was evaluated using the time-varying area under the curve (AUCt) (17) from Days 180–540 using the validation set.

**Figure 2:**
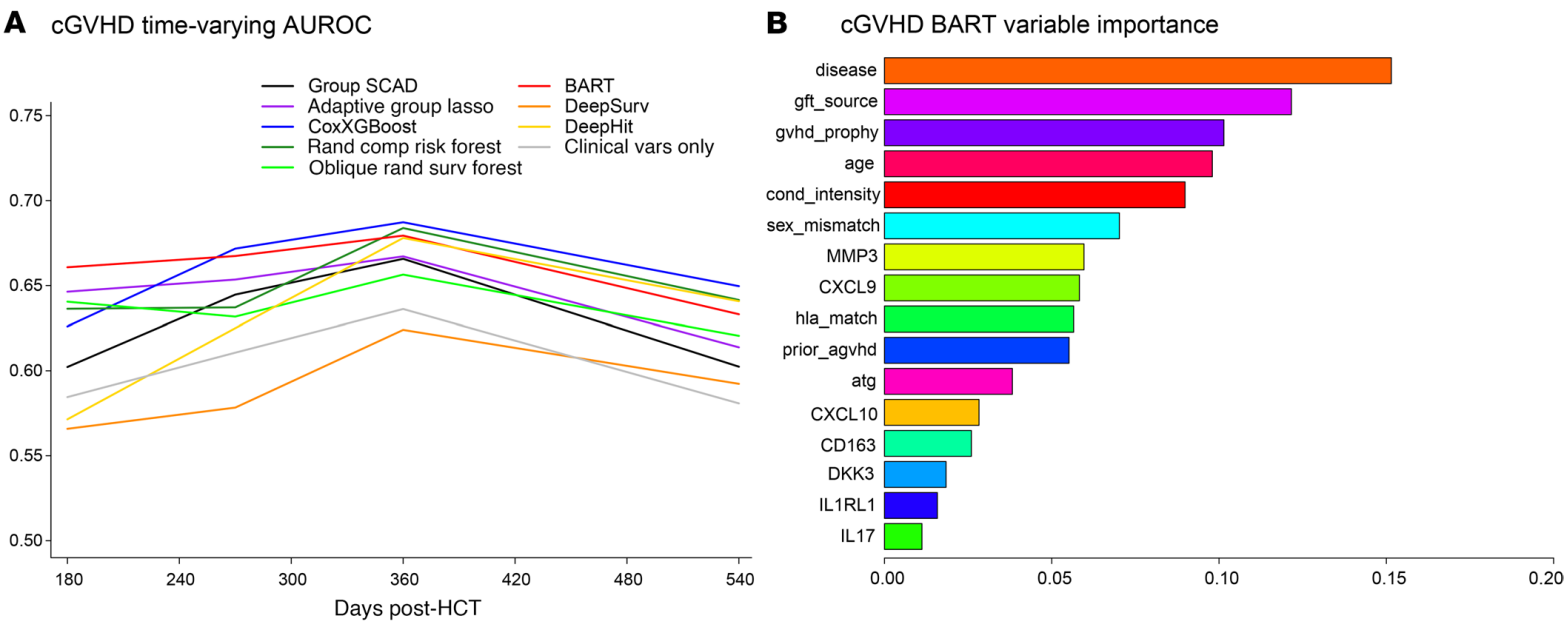
Dynamic risk prediction performance and key predictors in machine learning models of chronic GVHD. (**A**) Time-varying Area Under the ROC curve (AUCt) for cGVHD risk machine learning (ML) models with biomarkers and clinical variable effects. AUCt estimated at days 180, 270, 360, and 540 after HCT in the validation dataset. ML models were fitted using the training dataset. For calculation of AUCt at a given time point, patients with cGVHD onset through that time are classified as cases, while patients who are still alive are classified as controls. (**B**) BART variable importance metric for model of cGVHD with biomarkers and clinical variables. Importance metric describes how often each variable is used for splitting within the trees of the BART ensemble. These are Dirichlet posterior probabilities (see Methods).

The Group SCAD model with only clinical factors selected 4 clinical variables: age, graft source, F-M sex-mismatch, and prior aGVHD. Group SCAD models with biomarkers selected MMP3 and DKK3 while the Adaptive Group Lasso selected CXCL9, CXCL10, MMP3, DKK3, IL1RL1, and sCD163 (Supplemental Table 5). In the validation dataset, all ML methods with biomarkers other than DeepSurv provided higher AUCt for cGVHD compared with clinical factors only, suggesting that plasma proteins measured as early as Day 90/100 can inform underlying cGVHD that has not manifested clinically (Figure 2A and Supplemental Table 6). BART had the highest AUCt at Day 180, while CoxXGBoost had the highest AUCt at days 270, 360, and 540 after HCT; these were higher than the model with only clinical factors by greater than 5% across timepoints. Deep learning performed worse than other ML methods, possibly due to a limited sample size.

Since BART was the most consistent model across timepoints and had the highest performance for early evaluation, we assessed the variables’ importance in ML by examining BART’s posterior probabilities of selection. For cGVHD risk, variables with selection probabilities greater than 0.05, in order of ranking, were primary disease (malignant versus nonmalignant), graft source, GVHD prophylaxis, age, conditioning intensity, F-M sex mismatch, MMP3, CXCL9, HLA match, and prior aGVHD (Figure 2B).

### Multivariate ML models of moderate/severe cGVHD risk.

**Figure 3:**
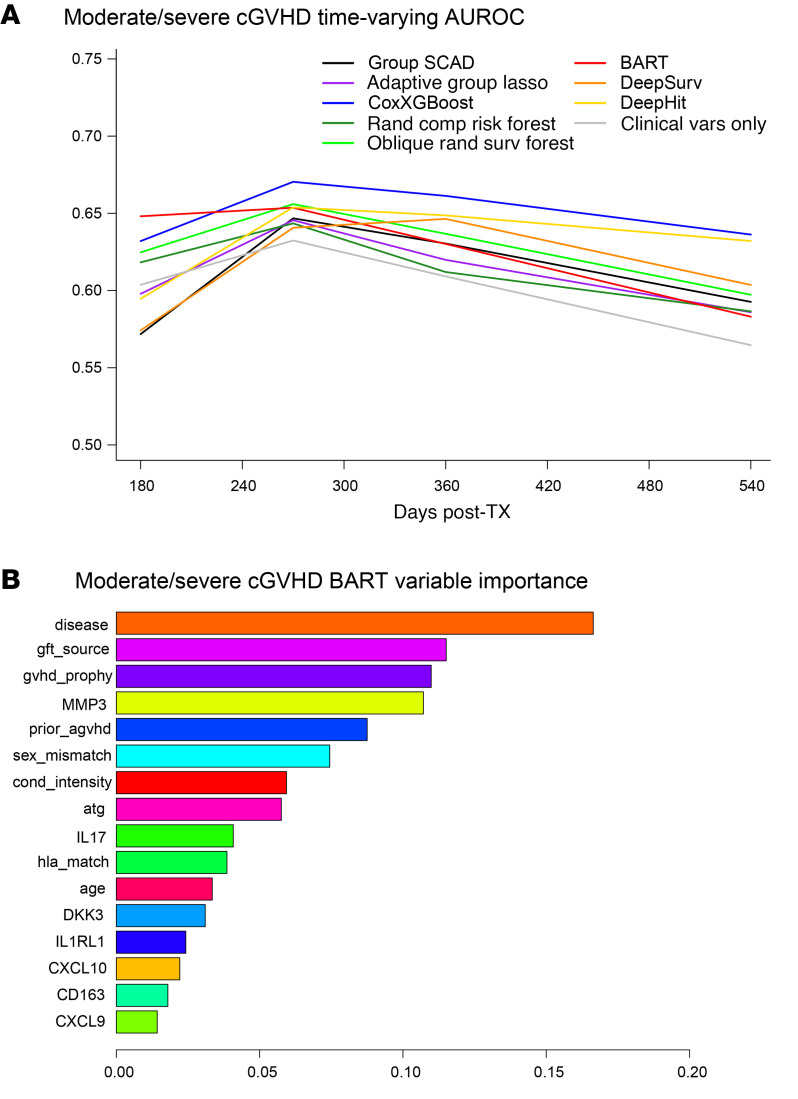
Dynamic risk prediction performance and key predictors in machine learning models of moderate/severe chronic GVHD. (**A**) Time-varying Area Under the ROC curve (AUCt) for moderate/severe cGVHD risk machine learning (ML) models with biomarkers and clinical variable effects. AUCt estimated at days 180, 270, 360, and 540 after HCT in the validation dataset. ML models were fitted using the training dataset. For calculation of AUCt at a given time point, patients with moderate/severe cGVHD onset through that time are classified as cases, while patients who are still alive are classified as controls. (**B**) BART variable importance metric for model of moderate/severe cGVHD with biomarkers and clinical variables. Importance metric describes how often each variable is used for splitting within the trees of the BART ensemble. These are Dirichlet posterior probabilities (see Methods).

For moderate/severe cGVHD, the Group SCAD model selected MMP3, graft source, and F-M sex-mismatch while Adaptive Group Lasso selected MMP3, DKK3, IL1RL1, graft source, primary disease, GVHD prophylaxis, and F-M sex-mismatch (Supplemental Table 7). In the validation dataset, all ML methods with biomarkers provided higher AUCt for moderate/severe cGVHD compared with Group SCAD with clinical factors only (Figure 3A and Supplemental Table 8). BART had the highest AUCt at Day 180 while CoxXGBoost had the highest AUCt at Days 270, 360, and 540; these were better than the model with only clinical factors by at least 4% at all time points. Deep learning approaches did not increase AUCt over other ML methods. The most commonly used clinical variables with selection probabilities greater than 0.05 in the BART model for moderate/severe cGVHD risk were the same as for overall cGVHD: primary disease, graft source, and GVHD prophylaxis; but, other important clinical variables differed from the overall cGVHD model and included prior aGVHD, F-M sex mismatch, conditioning intensity, and ATG. The ranking for the biomarkers also differed between outcomes with MMP3 having a higher probability of being greater than 0.05, IL17 lying near the threshold of 0.05, and CXCL9 ranked much lower as a predictor of moderate/severe cGVHD risk (Figure 3B).

### Multivariate ML models of NRM.

**Figure 4:**
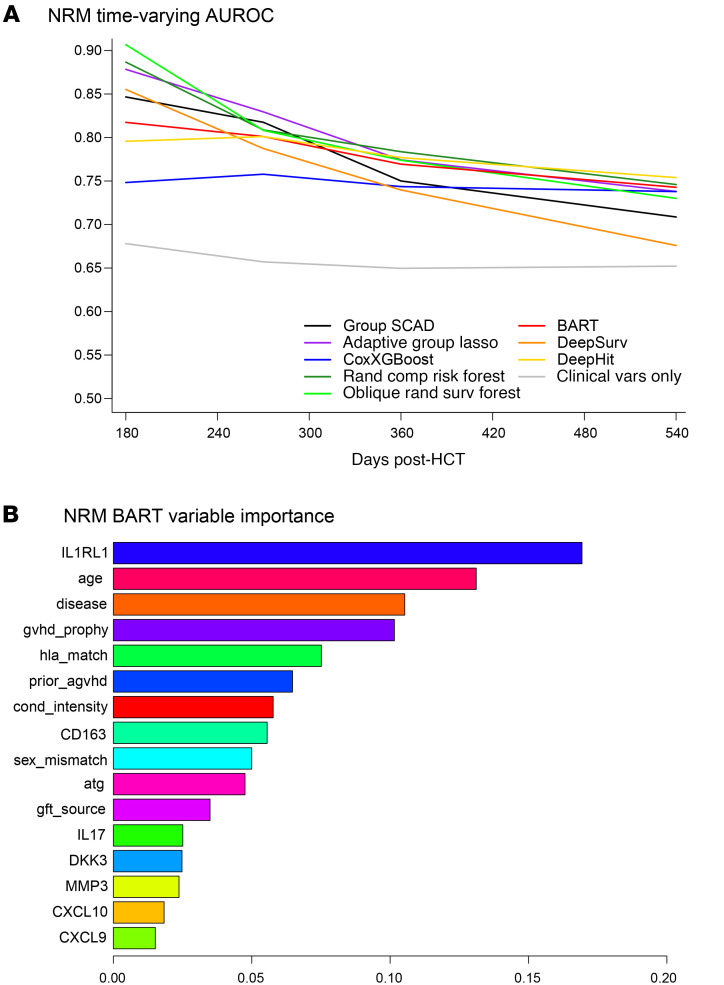
Dynamic risk prediction performance and key predictors in machine learning models of nonrelapse mortality. (**A**) Time-varying Area Under the ROC curve (AUCt) of Machine learning models for NRM using biomarkers and clinical variables. AUCt estimated at days 180, 270, 360, and 540 after HCT in the validation dataset. ML models were fitted using the training dataset. For calculation of AUCt at a given time point, patients with NRM through that time are classified as cases, while patients who are still alive are classified as controls. (**B**) BART variable importance metric for model of NRM with biomarkers and clinical variables. Importance metric describes how often each variable is used for splitting within the trees of the BART ensemble. These are Dirichlet posterior probabilities (see Methods).

For NRM, the Group SCAD model selected IL1RL1, MMP3, sCD163, age, and HLA-match. In addition to these, the Adaptive Group Lasso model also selected DKK3, graft source, primary disease, GVHD prophylaxis, and prior aGVHD (Supplemental Table 9). In the validation dataset, all ML methods for NRM prediction provided AUCt of 0.76–0.91 in early timepoints; these curves stayed high beyond day 270 after HCT compared with clinical factors only (Figure 4A and Supplemental Table 10). Adaptive Group Lasso, Group SCAD, BART, Random Competing Risks Forest, and Oblique Random Survival Forest yielded much higher AUCt than the model with only clinical factors. Deep learning approaches gave AUCt similar to but not superior to other ML models. The BART model provided the most consistent AUCt, and variables selected with probabilities greater than 0.05 were, in hierarchical order, IL1RL1, age, primary disease, GVHD prophylaxis, HLA-match, prior aGVHD, conditioning intensity, and sCD163 (Figure 4B). Of note, AUCt for Cox models of relapse for individual biomarkers adjusted for graft source are approximately 0.50, except for sCD163, which has an AUCt of 0.62 (Supplemental Figure 2).

### Prediction Metrics of BIOPREVENT models for cGVHD and NRM at day 360.

Because the BART models were consistently among the best ML methods in terms of AUCt, we further evaluated the BART-derived algorithms’ prediction performance. These models for cGVHD and NRM were further referred to as BIOPREVENT (BIOmarkers PREVENTion) models. Using the validation cohort, the BIOPREVENT models’ sensitivity, specificity, PPV, and NPV for the cumulative incidences of cGVHD and NRM at day 360 were evaluated at various cutpoints, where patients with estimated cumulative incidences at or above the threshold were predicted to have the event of interest (Supplemental Tables 11 and 12 and Supplemental Figures 3 and 4). We then determined the optimal cutpoint as the value that provided the best balance between sensitivity and specificity, ensuring the most effective trade off between true positive and true negative rates. For cGVHD, a cutpoint of 0.45 yielded a good balance across performance metrics with sensitivity and specificity of 64%, PPV of 58%, and NPV of 56% (Supplemental Table 11 and Supplemental Figure 3). A cutpoint for NRM incidence of 0.08 produced sensitivity of 70%, specificity of 69%, PPV of 14%, and NPV of 81% (Supplemental Table 12 and Supplemental Figure 4). Of note, the SCAD clinical variables only model had an optimal cutpoint of 1.52 for the cause-specific hazard ratio of cGVHD and resulted in a sensitivity and specificity of 58% while for NRM prediction, the clinical variables–only model had an optimal cutpoint of 1.63 and resulted in a sensitivity of 61% and specificity of 65% (Supplemental Table 13).

### BIOPREVENT models associate with increased risk of cGVHD and death without relapse.

**Figure 5:**
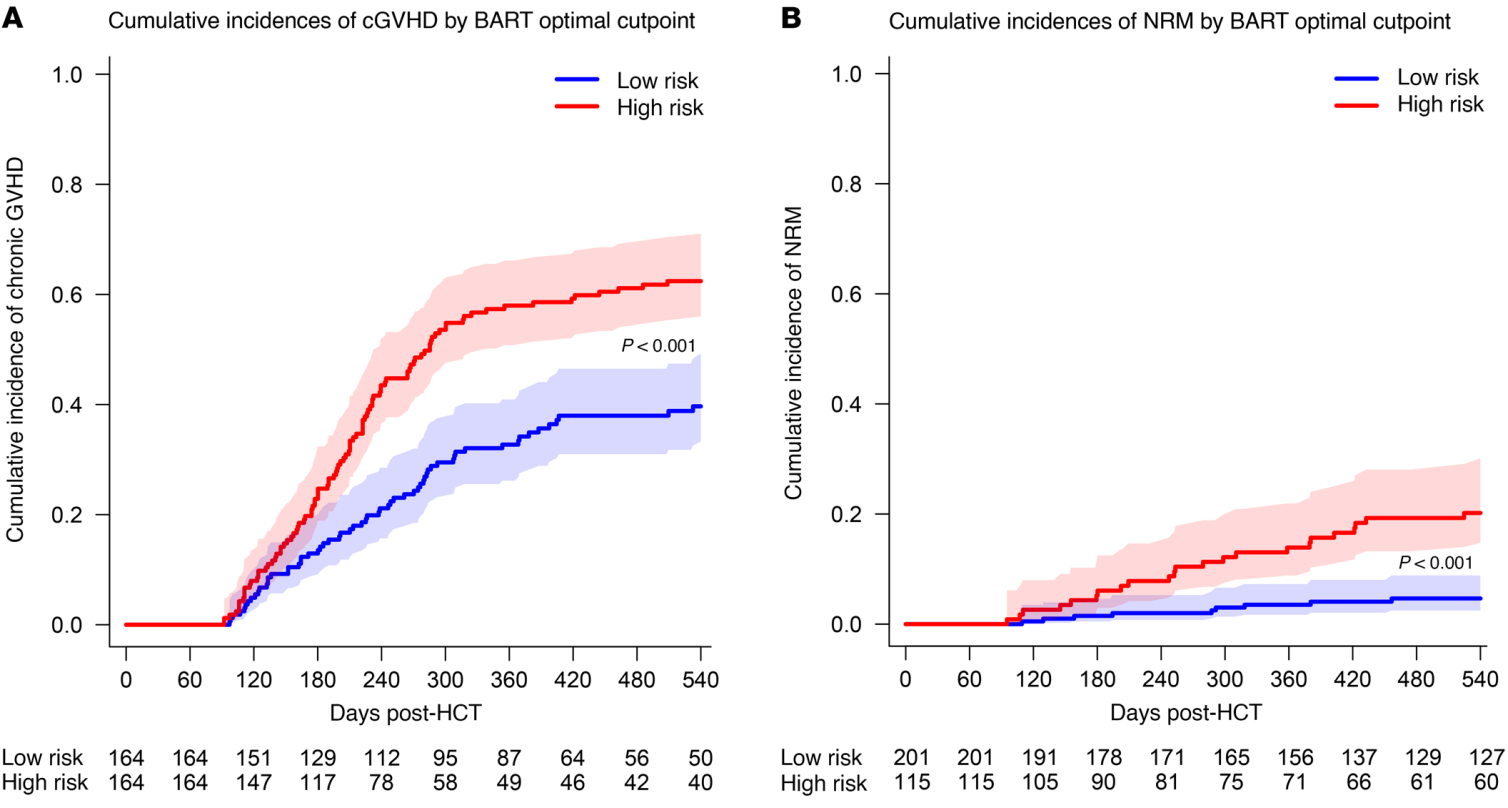
BIOPREVENT model–predicted risk groups discriminate chronic GVHD and nonrelapse mortality. (**A**) Cumulative incidences of cGVHD by BIOPREVENT model-predicted risk groups. Using the validation cohort, the BART model optimal cutpoint was selected based on the best balance between sensitivity, specificity, positive predictive value (PPV) and negative predictive value (NPV) for the cumulative incidences of cGVHD at Day 360 was of 0.45. A BART-predicted Day 360 cGVHD cumulative incidence cutpoint of 0.45 was used to classify patients into low and high risk groups. Gray’s test was used to compare cumulative incidence curves between risk groups. (**B**) Cumulative incidences of NRM by BIOPREVENT model–predicted risk groups. Using the validation cohort, the BART model–optimal cutpoint was selected based on the best balance between sensitivity, specificity, PPV and NPV for the cumulative incidences of NRM at Day 360. A BART-predicted day 360 NRM cumulative incidence cutpoint of 0.08 was used to classify patients into low and high risk groups. Gray’s test was used to compare cumulative incidence curves between risk groups.

We next asked whether the BIOPREVENT models could classify patients into low and high risk groups for clinical implementation. Based on the BART optimal cutpoint of 0.45 for cGVHD prediction, day 360 cumulative incidences of cGVHD were 58.0% in patients at/above this cutpoint vs. 32.7% in patients below this cutpoint (*P* < 0.0001, Figure 5A and Supplemental Table 14). Using the BART optimal cutpoint of 0.08 for NRM prediction, day 360 cumulative incidences of NRM in patients at/above and below this threshold were 13.9% and 3.5%, respectively (*P* < 0.0001, Figure 5B and Supplemental Table 15). Of note, the SCAD clinical variables–only model showed day 360 cumulative incidences of cGVHD of 51.8% in the high risk group versus 38.6% in the low risk group (Supplemental Figure 5A and Supplemental Table 16). Notably, the separation between the cumulative incidence curves is greater for the biomarker-plus-clinical predictors model than it is for the clinical predictors–only model. This is consistent with our prior findings on prediction performance, which is that the prediction models for cGVHD with both biomarkers and clinical variables account for a 6 point (on average) increase in AUC compared with the model with clinical variables only. For NRM prediction, the SCAD clinical variables–only model showed day 360 cumulative incidences of 12.4% in the high risk group versus 4.5% in the low risk group (Supplemental Figure 6B and Supplemental Table 17).

### BART prediction model calibration metrics.

**Figure 6:**
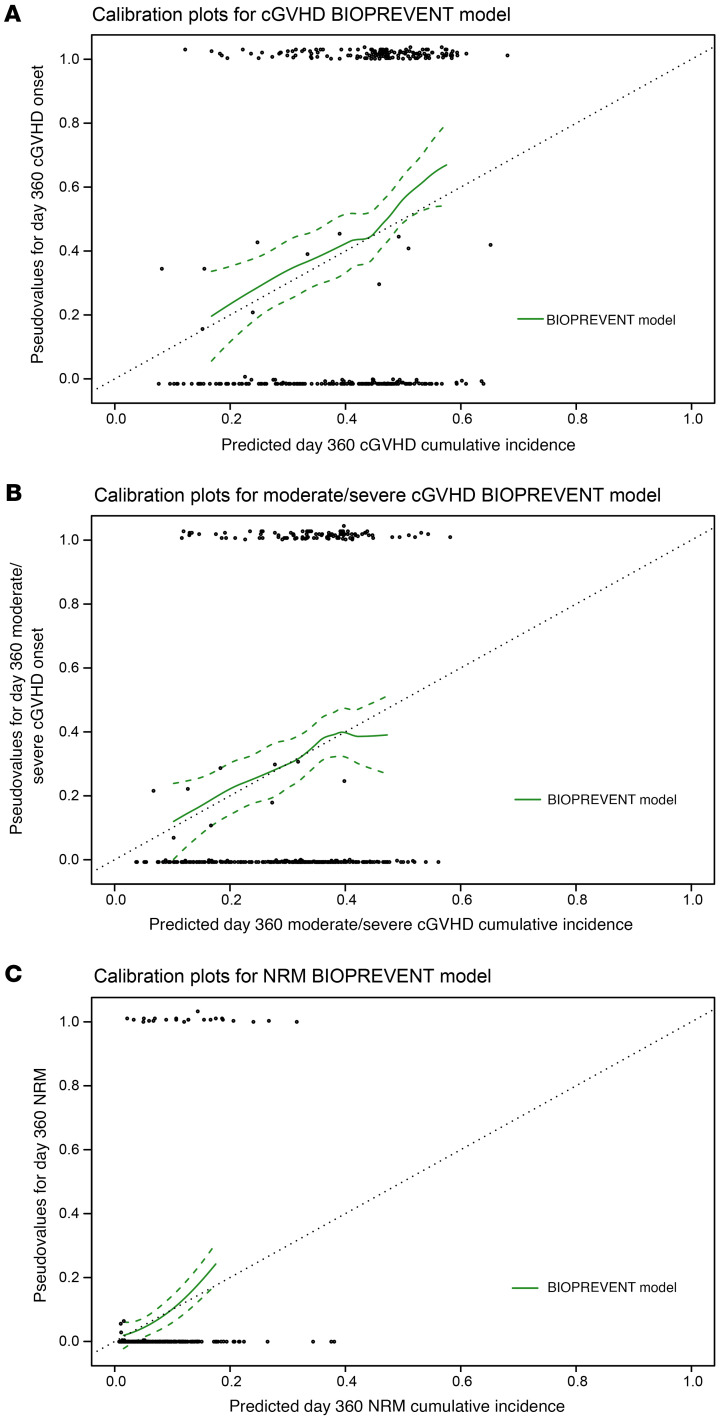
BIOPREVENT model shows good calibration for chronic GVHD and nonrelapse mortality. (**A**) BIOPREVENT model calibration plots for cGVHD at day 360. Pseudovalues and model predictions were computed using the validation dataset. Estimates of intercept and slope obtained from linear regression of pseudovalues of day 360 event status by BART model–predicted probability using the validation dataset shown in Supplemental Table 18. (**B**) BIOPREVENT model calibration plots for moderate/severe cGVHD at day 360. Pseudovalues and model predictions were computed using the validation dataset. Estimates of intercept and slope obtained from linear regression of pseudovalues of day 360 event status by BART model–predicted probability using the validation dataset shown in Supplemental Table 18. (**C**) BIOPREVENT model calibration plots for NRM at day 360. Pseudovalues and model predictions were computed using the validation dataset. Estimates of intercept and slope obtained from linear regression of pseudovalues of day 360 event status by BART model–predicted probability using the validation dataset shown in Supplemental Table 18.

We next constructed calibration curves that show how well the predicted probabilities of the BIOPREVENT model correspond with actual observed outcomes for the predictions of all grade cGVHD, moderate/severe cGVHD, and NRM at day 360, using the validation dataset. The LOESS smoothing curves indicate good calibration for these models, generally lying close to the 45˚ line of perfect calibration (Figure 6). The respective calibration slopes for all grade cGVHD, moderate/severe cGVHD, and NRM models were 1.080, 1.039, and 1.157 and did not differ significantly from 1; calibration intercepts were close to 0 for all models (Supplemental Table 18). Although the calibration curves indicate that the BART model demonstrates good accuracy in estimating individual risk probabilities, applying a fixed cutpoint to convert these probabilities into binary classifications (i.e., high risk versus low risk) introduces misclassification in the form of false positives and false negatives. To understand the factors contributing to these errors, we have now conducted a formal analysis of associations between baseline characteristics and the rates of false positives or false negatives. Patients who received bone marrow (BM) grafts experienced a higher rate of false negatives compared with those receiving PB grafts. This finding was driven by the lower incidence of cGVHD in patients receiving BM and lower predicted likelihood of cGVHD from the model, leading to only 7 cases above the cutpoint who are predicted to experience cGVHD. This finding underscores an important point: even when a risk prediction model is well calibrated at the population level, applying a binary threshold can still lead to systematic classification errors in subgroups with differing baseline risks, and thresholding of low versus high risk may need to be considered separately by subgroups.

### Web application of BIOPREVENT cGVHD and NRM.

Finally, we developed a publicly available web-application named BIOPREVENT cGVHD and NRM models that use BART-derived models to predict cGVHD and NRM risks for a given patient. This tool uses data on the 9 clinical variables and the 7 protein biomarkers at day 90/100 to make personalized estimates of the cumulative incidences of cGVHD and NRM through Day 540. BIOPREVENT cGVHD allows clinicians to access patient-specific predictions with no requirement of specialized knowledge in ML. The app features intuitive drop-down menus to enter the patient characteristics and biomarker values, with results displayed as cumulative incidence plots and tables for cGVHD and NRM up to 18 months after HCT. BIOPREVENT cGVHD can be accessed directly at https://bio-prevent.nmdp.org/ or through the CIBMTR website at https://cibmtr.org/CIBMTR/Resources/Research-Tools-Calculators

## Discussion

In this study, we evaluate whether machine learning methods can enhance the sensitivity and specificity of established cGVHD risk biomarkers. To be able to answer this question we have leveraged samples at days 90 or 100 after HCT and 7 plasma biomarkers previously validated with Cox regression analysis (8) in 4 cohorts totaling 1,310 HCT recipients in what is, to our knowledge, the largest biomarkers study for cGVHD prediction to date. Among the clinical variables, graft source was most often selected for predicting overall and moderate/severe cGVHD for the ML models where we examined the selection process for SCAD, Adaptive Group Lasso, and BART models. This finding was expected because recipients of BM grafts developed less cGVHD than PB graft recipients in a randomized trial (18) that was part of the current study. Related donor, primary disease, and age were selected by these models probably due to the fact that most patients with nonmalignant diseases are children and receive BM grafts. GVHD prophylaxis is also correlated with the graft source and selected as one of the top clinical variables. Other clinical variables were prioritized differently between overall cGVHD and moderate/severe cGVHD with prior aGVHD, F-M sex mismatch, conditioning intensity, and ATG being selected in priority for moderate/severe cGVHD risk.

For predicting overall cGVHD, CXCL9 and MMP3 were the most often selected. CXCL9 has been validated as a strong risk biomarker for cGVHD in several cohorts, particularly of adult recipients (8, 19, 20). It has been validated as a diagnostic and prognostic marker of severity in even more cohorts (19, 21–23). CXCL9 and CXCL10 are 2 chemokines that attract type 1 T cells expressing the receptor CXCR3^+^ (24). Furthermore, the presence of polymorphisms in CXCR3 ligands is a susceptibility marker for severe cGVHD (25). Since CXCL9 expression is upregulated in target tissues of cGVHD (e.g., skin, liver, lungs) in response to IFN-γ produced by donor-derived T cells, it acts as a chemoattractant for CXCR3^+^ effector T cells, promoting their migration into tissues, which contributes to the perpetuation of inflammation and tissue injury (26). High CXCL9 expression has also been associated with increased collagen deposition and fibrosis in skin fibrosing models through a CXCR3-dependent upregulation of col1a1 in fibroblasts (27) and pulmonary fibrosis (28). Targeting CXCR3/CXCL9 signaling is a potential therapeutic strategy to reduce T-cell infiltration and tissue damage. Several monoclonal antibodies and small molecules (MDX-1100, T487, SCH-546738, ACT-777991 (8a)) have been developed to block this pathway with unfortunately limited clinical efficacy at the exception of the emerging agent 8a in combination with an anti-CD3 antibody (29). In contrast, JAK inhibitors such as ruxolitinib has been FDA-approved for cGVHD treatment and have been shown to decrease CXCL9/CXCL10 levels in inflammatory diseases (30).

MMP3, also known as stromelysin-1, is a proteolytic enzyme that degrades the extracellular matrix during fibrosis and has been identified as a diagnostic biomarker in bronchiolitis obliterans (BOS) with higher concentrations compared with cGVHD without BOS (31). It was subsequently part of a panel for the diagnosis, prognosis, and risk of cGVHD (8, 19). MMP3 expression is upregulated by inflammatory cytokines such as TNFα, and IL-1β (32). MMP3 then degrades the extracellular matrix components, such as collagen, laminin, fibronectin, and proteoglycans, which contributes to fibrotic tissue remodeling. It has been specifically involved as a mediator of pulmonary fibrosis (33). MMP-3 can also activate other pro-MMPs, including MMP-1, MMP-7, and MMP-9, amplifying the matrix-degrading cascade (34). Since measurements in the current study were made 3 months after HCT, MMP3 elevation may indicate the cGVHD fibrosing process starts earlier than commonly considered. Therapeutic targeting of the fibrosing process of cGVHD is an unmet need. In autoimmune diseases, inhibition of the RIPK1 pathway with GSK2982772 decreased MMP3 production compared with the placebo group (35).

Along with CXCL9 and MMP3, IL-17 was selected as a biomarker for moderate/severe cGVHD risk; it is produced by Th17 cells and is associated with macrophage-driven inflammation and fibrosis in cGVHD (36). Although IL17 is typically challenging to measure in cGVHD plasmas, the use of an ultrasensitive immunoassay allowed its detection in patients who subsequently developed moderate/severe cGVHD. Selective ROCK2 inhibition with belumosudil downregulates proinflammatory cytokines like IL-17 and IL-21 and has been shown to reduce the number and activity of Th17 cells while promoting the expansion and function of regulatory T cells (37).

For NRM prediction, the biomarkers selected differed from cGVHD models, though most clinical features were similar. Age and primary disease were selected first, followed by GVHD prophylaxis, HLA-matching, prior aGVHD, and conditioning intensity. Importantly, among all clinical and biomarker variables, the protein IL1RL1 was selected first by all models. It has been associated with risk of aGVHD in several cohorts (38), and some cGVHD studies (19, 20). It has also consistently been associated with NRM (19, 38). Soluble IL1RL1 is the form measured by ELISA and is a decoy receptor of IL33 (39). It acts at several levels in the pathogenesis of cGVHD: (a) it interferes with the IL-33’s protective effects on regulatory T cells, type 2 innate lymphoid cells, and epithelial repair; (b) it is released when the endothelium is damaged, which has been observed in cGVHD (40, 41); and it is secreted by alloreactive T cells in preclinical GVHD models (42, 43). The murine anti-IL1RL1 that is full length has been shown to reduce alloreactivity in preclinical models by targeting the circulating excess of soluble IL1RL1 while maintaining IL1RL1+ regulatory cells in the tissues (42). Anti-human neutralizing IL1RL1 antibodies such as astegolimab are in phase II and III trials. Their safety and efficacy of astegolimab (a fully anti-human neutralizing ST2/IL1RL1 antibody) have been established in patients with COPD and adults with severe asthma (44, 45).

sCD163 was the second-most selected biomarker. It has been associated with cGVHD as early as 80 days after HCT (46) and recently with NRM (8). sCD163 is shed from the surface of inflammatory macrophages by the tumor necrosis factor-α–converting enzyme (TACE), also known as ADAM17 (47). Targeting monocyte/macrophage activation pathways represents an emerging therapeutic strategy. A recent phase II trial showed efficacy of colony-stimulating factor 1 receptor inhibition with axatilimab in recurrent/refractory cGVHD (48). Of note, in some preclinical models of GVHD, an unexpected neuroinflammation was observed (49), underscoring the need for careful consideration of the context of monocyte/macrophage inhibition. As CD163 is preferably expressed on activated macrophages, its inhibition might be more specific.

While the clinical and biomarker variables identified as the most important were generally consistent with prior work using standard approaches (8), the ML models showed improved prediction performance because ML offers greater flexibility through its ability to model nonlinear relationships between biomarkers and outcome and potential interactions between biomarkers and/or patient characteristics. The prediction models for cGVHD with both biomarkers and clinical variables account for a 6 point (on average) increase in AUC compared with the model with clinical variables only. The models for NRM with both biomarkers and clinical variables account for a 12 point (on average) increase in AUC compared with the clinical factors–only prediction model. The BART-derived models consistently provided the highest AUCt for prediction of future cGVHD and NRM. Our ML models containing biomarkers showed better AUROC at 0.76–0.91 versus 0.62 in contrast to ML models trained from 33,927 patients using only clinical characteristics in the EBMT-TCWP study (50), suggesting that addition of proteomic biomarkers strengthens the accuracy of the AUROC. Our study presents the distribution of predictive scores and statistical performances for ML models in cGVHD. The risk scores from the BART-derived ML models may help stratify patients at low and high risk for cGVHD/NRM who may benefit from additional monitoring and future preemptive intervention.

In our study, we explore the use of deep learning for GVHD evaluation. Unfortunately, deep learning models performed, at best, similar to the other ML methods considered, which may be due to an inability of the former’s complex neural network structure to effectively learn the relationships between biomarkers and cGVHD risk without a much larger sample size, in the tens of thousands, as it has been done for diagnosing lung cancer with imaging (51).

Generalizability of statistical algorithms for GVHD care have been insufficient (52). Most ML algorithms developed for HCT have been evaluated only on the data they were trained on; studying their performance metrics on a validation set permits a proper assessment of how well the algorithms generalize to unseen data (53). Applications have been made available for HLA matching and immune suppression discontinuation (54, 55), but a tool using ML in HCT has yet to be published. We offer the BIOPREVENT cGVHD application that calculates individualized scores based on clinical and biomarker data, trained on the largest-scale multicenter dataset with cGVHD biomarkers to date. Indeed, we have trained and validated the biomarker-based predictive tool in a diverse population across multiple trials, transplant centers, and data sources, with a wide range of GVHD prophylaxis regimens, conditioning intensities, and patient-specific risk factors. This supports its real world utility for patient-specific risk prediction in a heterogeneous setting. The real-world utility of the predictive tool comes from the potential to tailor treatment strategies to patients based on their cGVHD risk. To test the value of intervention based on our biomarker-based predictive tool, a randomized study will need to be developed where patients are randomized to an intervention based on the risk of the patients. For high-risk patients, one could consider randomizing to addition of preemptive immunosuppressive therapy, such as ruxolitinib versus placebo. For low-risk patients, rather than adding immunosuppression, which can increase the risk of relapse or infection, the biomarker-based predictive tool can be useful in guiding immunosuppression discontinuation decisions. Here, clinicians may be more interested in randomizing between a rapid immunosuppression taper versus a standard immunosuppression taper. Note that studying preemptive therapy in a high-risk population can lead to important reductions in required sample sizes, since the required sample size for detecting a difference in incidences of events is inversely proportional to the event rate in the study population. Tailoring and studying treatment strategies based on biomarker-based predictive tools are extremely useful real-world applications. To illustrate this, we performed 2 power calculations for a hypothetical trial using either an enriched high-risk group based on the BIOPREVENT algorithm or an all-comers group. We assumed that the day 540 cumulative incidence of chronic GVHD in the control group, starting at a day 90 landmark time, is 49.5% for the full HCT population and 63.3% for the high risk population, based on the observed rates in our current cohort. We also assumed follow up of 450 days for all patients in the trial, corresponding with follow up between the day 90 landmark and day 540 (approximately 1.5 years) after HCT. The sample size for 80% power to detect a subdistribution hazard ratio of 0.60 between treatment and control, using Gray’s test, would be 290 for the all-comers population or 222 for the high-risk group.

However, there are limitations to this study. While ML-based approaches can offer unbiased feature selection, 2 major challenges are understanding how they function and determining how influential variables impact the outcome of interest. That said, the BIOPREVENT cGVHD application will provide risk scores for cGVHD and NRM for each patient based on their clinical characteristics and Day 90/100 biomarkers. Another limitation of the study is that only 1 sample was tested due to the lack of additional longitudinal samples collected after day 100 in these cohorts. Increasing the number of biomarker timepoints could increase AUROC, as was shown for aGVHD prediction with a dynamic probabilistic algorithm, daGOAT (56). We chose to include only 7 previously validated risk biomarkers of cGVHD, but adding more markers including cellular subsets, such as regulatory T cells, T follicular helper cells, and T follicular regulatory cells, Th17-prone CD146^+^ T cells that have all been positively or negatively correlated with cGVHD (57–59) could increase the accuracy of the model. However, paired plasma and PBMCs are not readily available in most biobanks, and the low throughput of cellular measurements preclude timely generation of large amount of cellular datapoints necessary for ML models. Furthermore, adding many predictors without increasing the sample size can adversely impact ML models by introducing collinearity or noise (60). Prior acute GVHD was included in the ML models and was a variable picked by the BART model. However, some other potential important variables (late acute GVHD, immunosuppression status including modulation for increased risk of relapse at the time of sampling) could not be included because only retrospective deidentified defined data sets were available to develop prediction models and it was, therefore, not possible to go back to the centers to verify these variables. The models for NRM prediction treat relapse as a competing risk. Finally, PTCY has shown lower cGVHD incidence than CNI-based prophylaxis (61, 62). Although patients receiving PTCY represented a low number in our study (*n* = 27), PTCY was included in the variables compared in GVHD prophylaxis and was picked as a variable in the Group Lasso where hazard ratios are calculated for each variable (see Supplemental Table 5). Thus, the interpretation of the current algorithm in PTCY GVHD prophylaxis is still uncertain and the model will need to be validated on larger number of samples from patients receiving PTCY. We speculate that the BIOPREVENT model will work with PTCY prophylaxis since, once present, the biology of cGVHD should be relatively similar in patients receiving PTCY GVHD prophylaxis and patients receiving CNI-based GVHD prophylaxis. For example, tree-based machine learning using clinical, cellular, and proteomic variables in samples at day 28 after HCT from patients receiving PTCY showed that CXCL9 predicted acute GVHD (63) and we suspect that several of the 7 proteins measured at day 90 after HCT will also be selected in our machine learning models.

In summary, early identification of patients who will develop cGVHD may permit more stringent monitoring and preemptive interventions. These data support future research to further validate and implement these ML algorithms into the clinic.

## Methods

### Sex as a biological variable.

Both donor and recipient sex were included as biological variables in this HCT study. Because donor-recipient sex combinations are known to influence cGVHD risk — particularly female-to-male transplants, where alloimmune responses to H-Y antigens may increase susceptibility — sex was treated as an important clinical covariate in all analyses. All transplant pairs, regardless of sex, were enrolled, and donor-to-recipient sex matching was systematically captured to account for its potential impact on cGVHD outcomes.

### BMTCTN 0201 cohort.

295 HCT recipients of unrelated donor transplantation with a day 90 post-HCT sample were included in this cohort. 153 patients received PB grafts and 142 patients received BM grafts. Patients who developed cGVHD before or on day 90 were excluded. Maximum cGVHD severity was graded by the limited and extensive score. Patients with relapse were included in the analysis.

### BMTCTN 1202 cohort.

641 HCT recipients from a contemporary cohort with samples available at day 90 after HCT were included. 513 patients received PB grafts, and 128 patients received BM grafts. Patients who developed cGVHD before or on day 90 were excluded. Maximum cGVHD severity was graded by the NIH global scoring system (5). Patients with relapse were included in the analysis. Other exclusion criteria to remove potential confounding factors for cGVHD development were applied: (a) patients who received T cell depletion (ATG, Alemtuzumab), (b) patients who received post-transplant cyclophosphamide, (c) patients who received cord grafts; (d) patients who developed acute GVHD (aGVHD) or received more than 1mg/kg corticosteroids after day 56 post-HCT.

### DFCI cohort.

99 HCT recipients with day 100 post-HCT samples available in the Paquarello Tissue Bank for Hematologic Malignancies were included. 74 patients received PB grafts, and 25 patients received BM grafts. Patients who developed cGVHD before or on day 90 were excluded. Maximum cGVHD severity was graded by the NIH global scoring system (5). Patients with relapse were included in the analysis. Other exclusion criteria to remove potential confounding factors for cGVHD development were applied: (i) patients who received T cell depletion (ATG, Alemtuzumab), (ii) patients who received post-transplant cyclophosphamide, (iii) patients who received cord grafts; (iv) patients who developed aGVHD.

### Multicenter pediatric/adults NCT02194439 cohort.

275 HCT recipients (203 aged ≤ 20 years) with samples available at day 100 after HCT were included. 89 patients received PB grafts, and 186 patients received BM grafts. Patients who developed cGVHD before or on day 90 were excluded. Maximum cGVHD severity was graded by the NIH global scoring system (5). Other exclusion criteria to remove potential confounding factors for cGVHD development were applied, including removing patients who received cord grafts.

### Enzyme-linked immunosorbent assay.

ELISA was performed blinded from clinical information and reference standard results. ELISA procedures and parameters have previously been described (8). Supplemental Table 2 provides details of the ELISA kits including catalog numbers.

### BIOPREVENT cGVHD shiny application.

The BIOPREVENT (BIOmarkers PREVENTion model) cGVHD interactive web application was created using the shiny R package (64). The app features drop-down menus for the user to enter 9 clinical characteristics and 7 biomarker values for a transplant recipient. After all values are entered, the user can click a button to generate patient-specific predictions for chronic GVHD and nonrelapse mortality. Results are displayed as a cumulative incidence plot and table of predicted probabilities and 95% credible intervals for 3 to 18 months after transplant in half-month increments. After the predictions are generated, the user can download a report in the form of an html R markdown document that includes all cumulative incidence plots and tables and a summary of the user input patient characteristics. The application can be accessed directly at https://bio-prevent.nmdp.org/ or through the CIBMTR website at https://cibmtr.org/CIBMTR/Resources/Research-Tools-Calculators

### Statistics.

Patient characteristics were described by frequencies and percentages for categorical variables and by medians and ranges for continuous variables. Associations between biomarkers were described using the Spearman rank correlation. For statistical models, missing values of each biomarker were imputed using the marker’s median value among nonmissing observations. Cox proportional hazards regression models were used to evaluate univariable associations of biomarkers with the cause specific hazards of overall cGVHD, moderate/severe cGVHD, NRM, and relapse. For ML analysis, the study population was randomly split into training and validation cohorts comprising 75% and 25% of the patients, respectively. ML models for cGVHD and NRM were constructed using Cox regression with Group Smoothly Clipped Absolute Deviation (SCAD) (9) and Adaptive Group Lasso (10) penalties, Cox regression with extreme gradient boosting (CoxXGBoost) (11), Random Competing Risks Forests (12), Oblique Random Survival Forests (13), Bayesian Additive Regression Trees (BART) (14), and deep learning via DeepSurv (15) and DeepHit (16). Deep learning constitutes the subset of ML models that employ deep neural networks. Predictors included in the ML models are 7 biomarkers, IL1RL1, IL17, CXCL9, CXCL10, MMP3, DKK3, and soluble (s)CD163, and 9 clinical variables, age, graft source, primary disease, HLA-match, conditioning, GVHD prophylaxis, ATG, female-to-male donor-recipient (F-M) sex mismatch, and prior aGVHD. To reduce skewness, biomarker levels were transformed via the natural logarithm for modeling. All models of cGVHD onset, NRM, and relapse were landmark analyses at day 90/100 including patients still at risk for the event.

Each ML model was constructed using the training set; its predictive accuracy was evaluated using the time-varying area under the curve (AUCt) (17) from days 180–540 using the validation set. The sensitivity, specificity, positive predictive value (PPV), and negative predictive value (NPV) of each model was also assessed. Variable importance was evaluated using the BART models’ Dirichlet posterior mean splitting probabilities, which describe how frequently each variable is used in the BART tree ensemble. Model calibration was evaluated by plotting pseudovalues of cGVHD and NRM status versus their model-predicted probabilities and fitting LOWESS smoothing curves to the values; the slopes and intercepts for linear models of pseudovalues regressed on predicted probabilities were also estimated.

### Study approval.

The 4 cohorts were previously approved by Institutional Review boards of all BMTCTN participating centers, of Dana Farber Cancer Institute and 6 pediatric centers: Children’s National Medical Center, Texas Children’s Hospital, Fred Hutchinson Cancer Center, Boston Children’s/Dana Farber Cancer Institute, Johns Hopkins, and Indiana University School of Medicine. Informed consent was obtained from all patients or their legal guardians before HCT and sample collection.

### Data availability.

Biomarkers raw data are available through a material transfer agreement with MUSC, and all direct inquiries should be addressed to SP. All detection tools are available through commercial vendors. All data associated with this paper are present in the paper and/or in the Supplemental Materials and/or in the Supporting Data Values file. The BIOPREVENT cGVHD application can be accessed at https://bio-prevent.nmdp.org/

## Author contributions

MJM, BRL, and SP designed the study and wrote the main manuscript text; DD performed proteomics experiments; YY compiled clinical and biomarkers data; LER constructed the BIOPREVENT cGVHD webapp; and JR built and maintained the Pasquarello tissue bank at DFCI. All authors wrote the manuscript.

## Funding support

This work is the result of NIH funding, in whole or in part, and is subject to the NIH Public Access Policy. Through acceptance of this federal funding, the NIH has been given a right to make the work publicly available in PubMed Central.

- R01CA264921 (to SP, BRL).
- US-National Cancer Institute.
- U10HL069294.
- U24HL138660.
- R01HD074587.
- Pasquarello Tissue Bank for Hematologic Malignancies.
- P01HL158505.

## Supplementary Material

Supplemental data

ICMJE disclosure forms

Supporting data values

## Figures and Tables

**Table 1:**
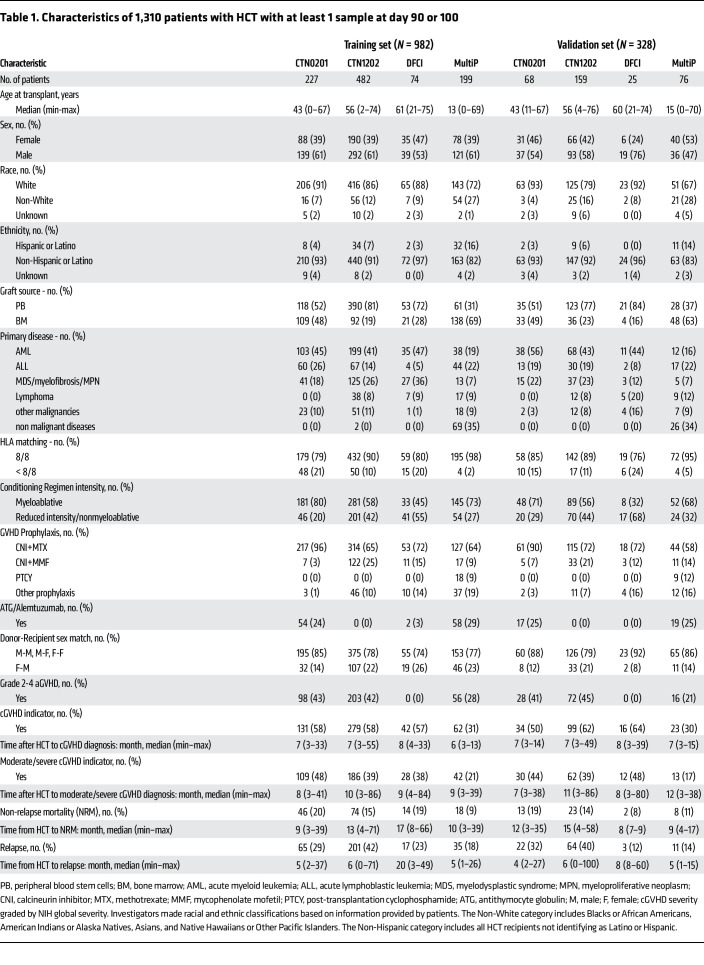
Characteristics of 1,310 patients with HCT with at least 1 sample at day 90 or 100

## Version 1 02/16/2026

Electronic publication
