# Supplementary material for: The BIOPREVENT machine-learning algorithm predicts chronic graft-versus-host disease and mortality risk using posttransplant biomarkers
Source: J Clin Invest. 2026 Feb 16;136(4):e195228. doi: 10.1172/JCI195228 (PMC12904722; doi:10.1172/JCI195228)
Supplement: Supplemental data [file jci-136-195228-s292.pdf]

SUPPLEMENTAL DATA

The BIOPREVENT machine learning algorithm predicts chronic graft-versus-host disease and mortality risk using post-transplant biomarkers

Michael J. Martens,[1,2] Debjani Dutta,[3,4] Yongzi Yu,[2] Lisa E. Rein,[1] Jerome Ritz,[5] Brent R. Logan,[1,2] and Sophie Paczesny[3,4]

Table of Contents

Supplemental Figure 1. Spearman correlation of marker concentrations at Day 90/100 post-HCT in the full cohort
Supplemental Figure 2. Time-varying area under the ROC curve (AUCt) for Cox models of relapse with biomarkers adjusted for graft source
Supplemental Figure 3. BART models' sensitivity, specificity, positive predictive value (PPV), and negative predictive value (NPV) for cumulative incidences of cGVHD at Day 360 at various cutpoints for the model-estimated cumulative incidence
Supplemental Figure 4. BART models' sensitivity, specificity, positive predictive value (PPV), and negative predictive value (NPV) for cumulative incidences of NRM at Day 360 at various cutpoints for the model-estimated cumulative incidence
Supplemental Figure 5. Cumulative Incidence Estimates of Risk Groups Determined by SCAD Clinical Variables Only Model

Supplemental Table 1. Frequencies of primary and secondary outcomes in the full cohort and separated by training and validation sets
Supplemental Table 2. Details of ELISA kits used for biomarkers measurements
Supplemental Table 3. Biomarker levels at Day 90/100 post HCT from the full cohort
Supplemental Table 4. Univariate Cox regression models of each biomarker's effect on cGVHD
Supplemental Table 5. Penalized regression models of cGVHD with biomarkers and clinical variables
Supplemental Table 6. Time-varying area under the ROC curve (AUCt) for machine learning models of cGVHD with biomarkers and clinical variables
Supplemental Table 7. Penalized regression models of moderate/severe cGVHD with biomarkers and clinical variables
Supplemental Table 8. Time-varying area under the ROC curve (AUCt) for machine learning models of moderate/severe cGVHD with biomarkers and clinical variables
Supplemental Table 9. Penalized regression models of NRM with biomarkers and clinical variables

Supplemental Table 10. Time-varying area under the ROC curve (AUCt) for machine learning models of NRM with biomarkers and clinical variables

Supplemental Table 11. BART cGVHD model prediction metrics

Supplemental Table 12. BART NRM model prediction metrics

Supplemental Table 13. SCAD clinical only model prediction metrics for cGVHD and NRM at the optimal cutpoint

Supplemental Table 14. Cumulative incidence of cGVHD by BART model-predicted risk groups

Supplemental Table 15. Cumulative incidence of NRM by BART model-predicted risk groups

Supplemental Table 16. Cumulative incidence of cGVHD by SCAD Clinical Variables Only model-predicted risk groups

Supplemental Table 17. Cumulative incidence of NRM by SCAD Clinical Variables Only model-predicted risk groups

Supplemental Table 18. BART prediction model calibration metrics

Supplemental Figure 1. Spearman correlation of marker concentrations at Day 90/100 post-HCT in the full cohort

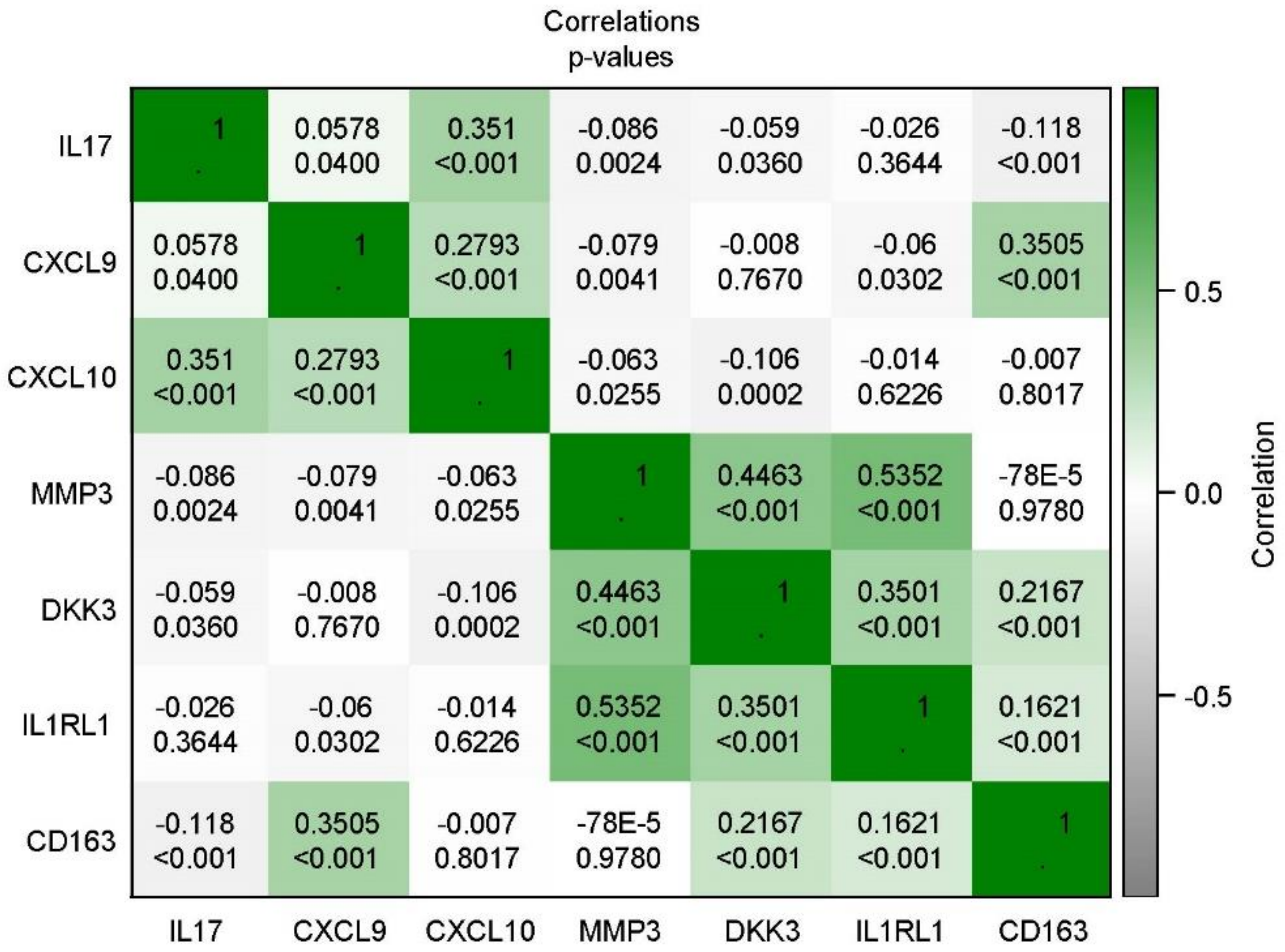

Supplemental Figure 2. Time-varying Area Under the ROC curve (AUCt) for Cox models of relapse with biomarkers adjusted for graft source

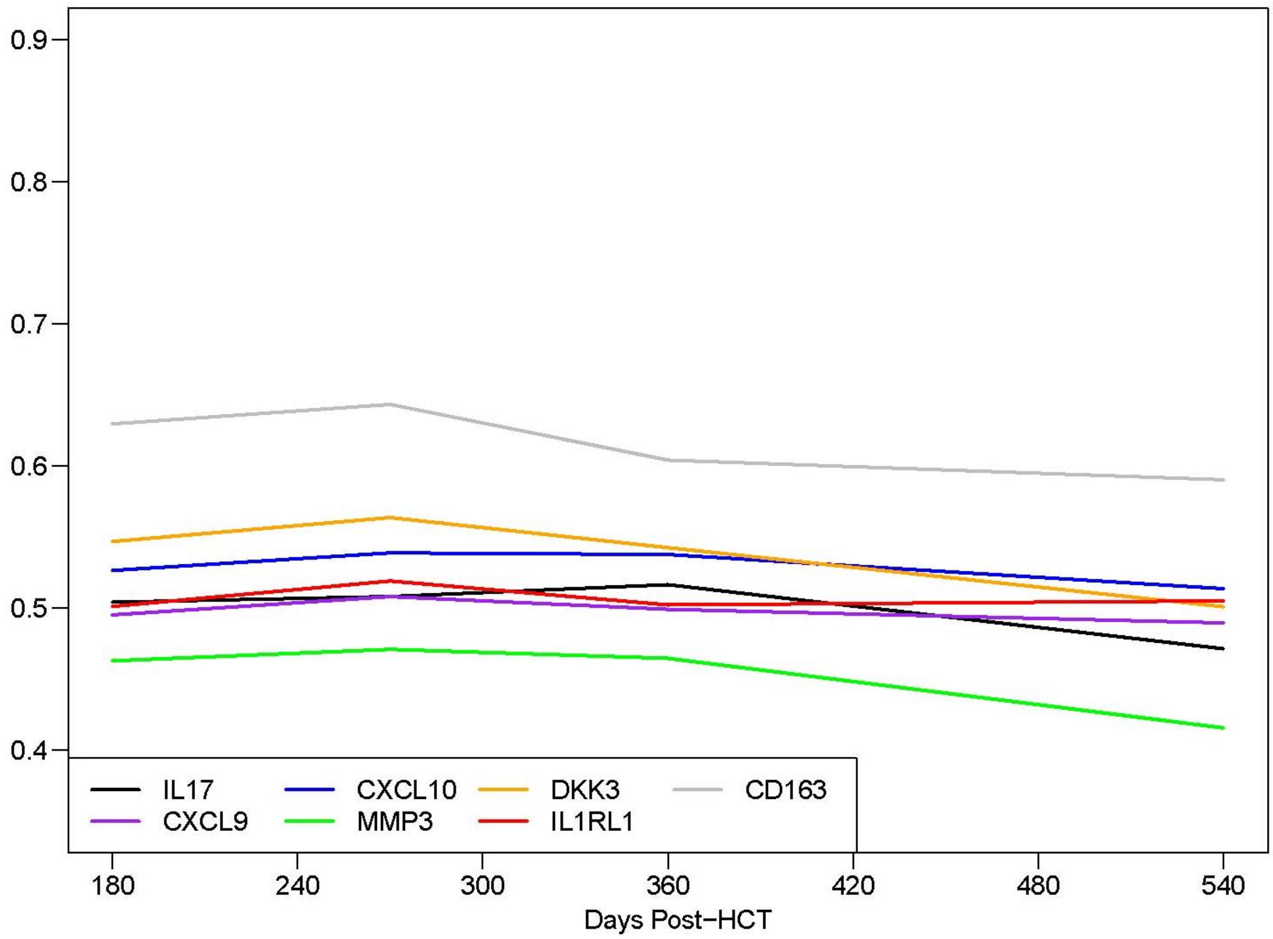

Supplemental Figure 3. BART models' sensitivity, specificity, positive predictive value (PPV), and negative predictive value (NPV) for cumulative incidences of cGVHD at Day 360 at various cutpoints for the model-estimated cumulative incidence

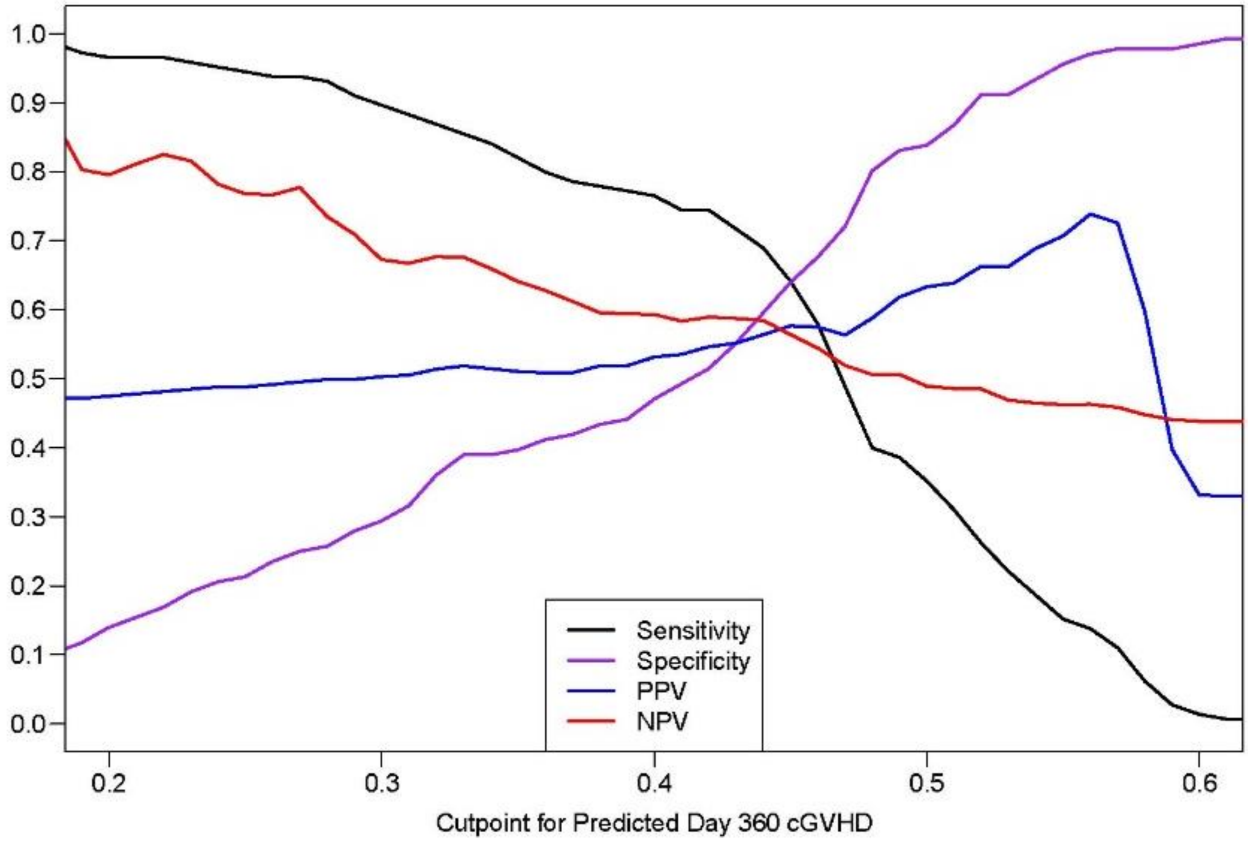

Supplemental Figure 4. BART models' sensitivity, specificity, positive predictive value (PPV), and negative predictive value (NPV) for cumulative incidences of NRM at Day 360 at various cutpoints for the model-estimated cumulative incidence

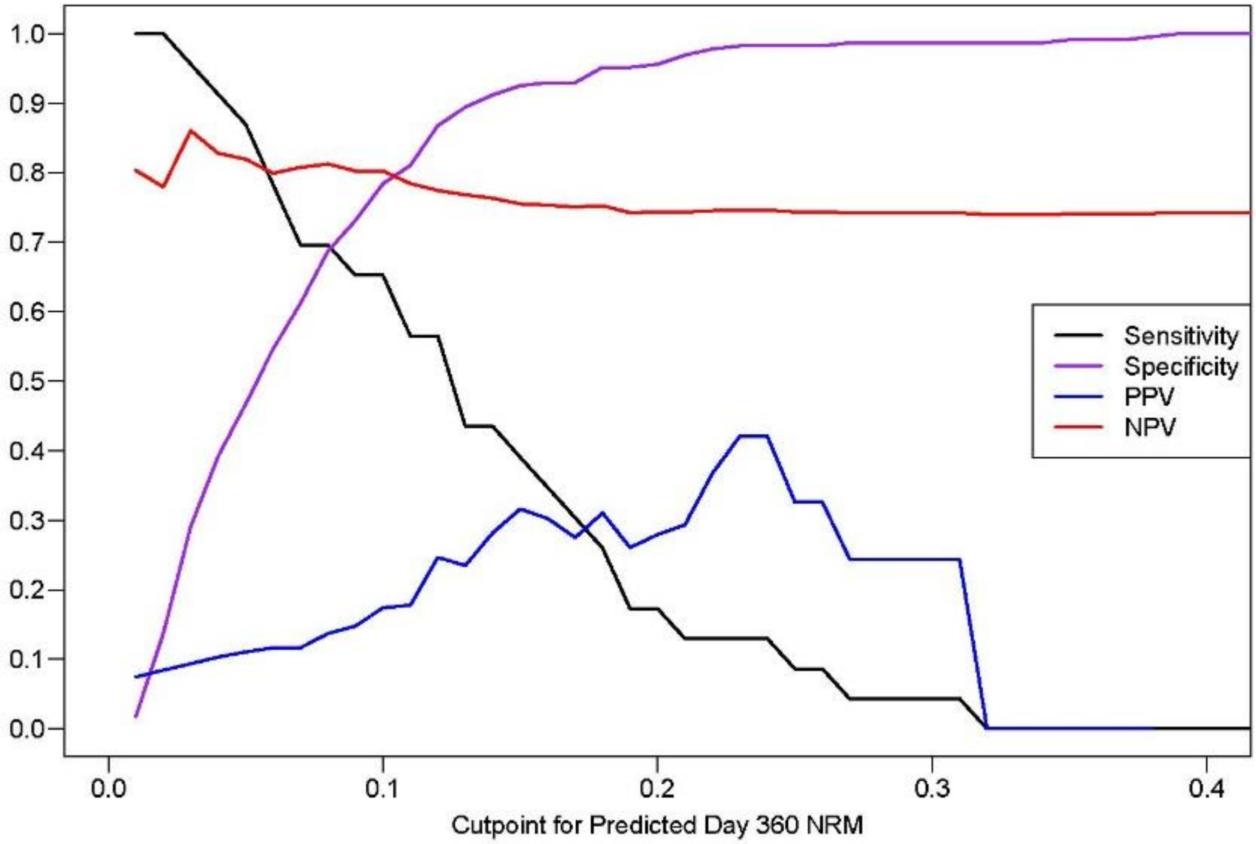

Supplemental Figure 5. Cumulative Incidence Estimates of Risk Groups Determined by SCAD Clinical Variables Only Model

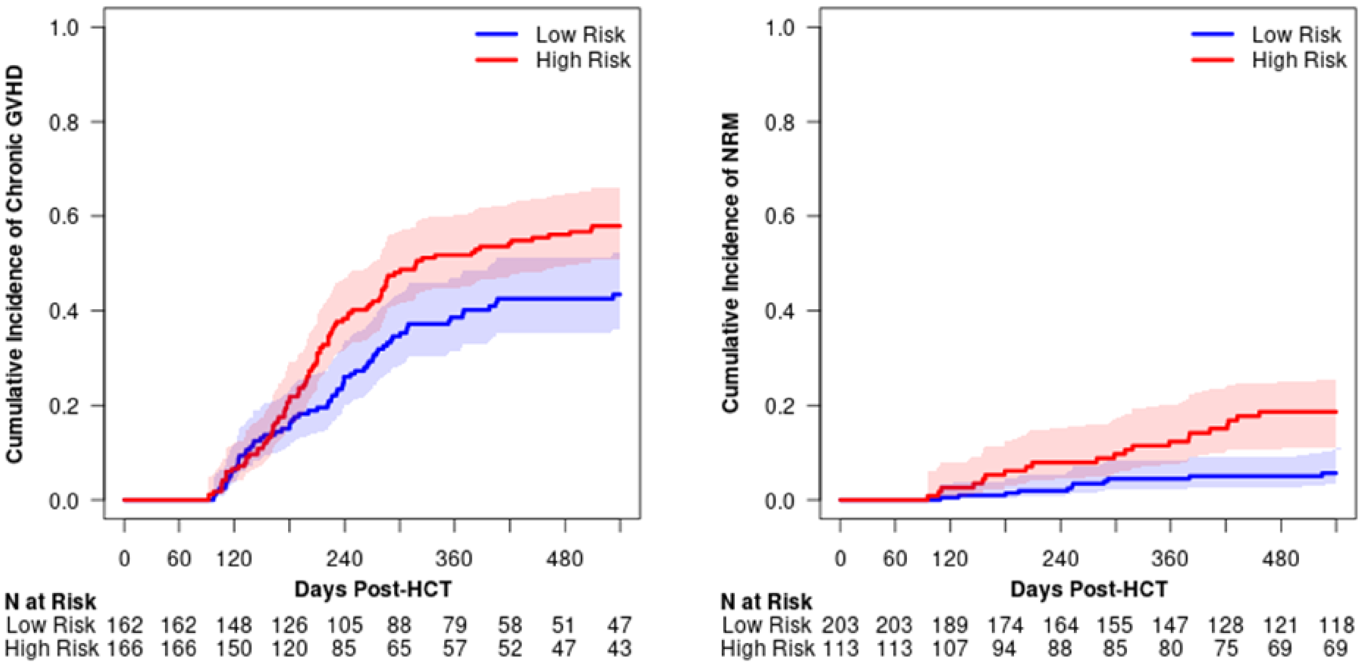

Supplemental Table 1. Frequencies of primary and secondary outcomes in the full cohort and separated by training and validation sets

Outcome	Training Set (N=982)	Validation Set (N=328)	All Patients (N=1310)
**cGVHD, n (%)**	514 (52.3%)	172 (52.4%)	686 (52.4%)
**Moderate/severe cGVHD, n (%)**	365 (37.2%)	117 (35.7%)	482 (36.8%)
**NRM, n (%)**	152 (15.5%)	46 (14.0%)	198 (15.1%)
**Relapse, n (%)[1]**	246 / 848 (29.0%)	80 / 292 (27.4%)	326 / 1140 (28.6%)

[1] Only patients with malignant disease are included in denominator

Supplemental Table 2. Details of ELISA kits used for biomarkers measurements

Protein name	Description	Commercial provider	Catalog #	Plasma dilution	LLOD	ULOD
IL1RL1	Interleukin-1 receptor-like 1, IL-33 receptor, Stimulation-2	R&D/bio-techne Quantikine	DST200	1:50	31 pg/ml	2000 pg/ml
IL17	interleukin 17	R&D/bio-techne Quantikine	HS170	undiluted	0.3 pg/ml	15 pg/ml
CXCL9	C-X-C motif chemokine ligand 9	RayBiotech	ELH-MIG	1:20	8 pg/ml	6000 pg/ml
CXCL10	C-X-C motif chemokine ligand 10	R&D/bio-techne Quantikine	DIP100	1:20	8 pg/ml	1000 pg/ml
MMP3	matrix metallopeptidase 3	R&D/bio-techne Duoset	DY513-05	1:25	31 pg/ml	2000 pg/ml
DKK3	dickkopf WNT signaling pathway inhibitor 3	R&D/bio-techne Duoset	DY1118	1:50	18 pg/ml	2500 pg/ml
CD163	CD163 molecule	R&D/bio-techne Quantikine	DC1630	1:10	1.5 ng/ml	100 ng/ml

Supplemental Table 3. Biomarker levels at day 90/100 post HCT from the full cohort

Biomarker	Units	N	N Miss	Minimum	Lower Quartile	Mean	Median	Upper Quartile	Maximum
IL17	pg/ml	1263	47	0.01	0.05	1.11	0.48	1.25	15.66
CXCL9	ng/ml	1310	0	0.00	0.61	3.75	1.84	4.00	89.63
CXCL10	ng/ml	1263	47	0.00	0.16	1.21	0.43	0.83	13.74
MMP3	ng/ml	1310	0	0.00	6.00	27.78	12.00	31.00	1798.00
DKK3	ng/ml	1260	50	9.39	44.00	66.74	57.00	75.18	1368.00
IL1RL1	ng/ml	1310	0	2.00	14.00	37.86	21.00	40.08	303.00
CD163	ng/ml	1263	47	131.09	508.12	702.92	683.23	901.04	1687.73

Supplemental Table 4. Univariate Cox regression models of each biomarker's effect on cGVHD

Biomarker	Effect[1]	Hazard Ratio	95% Lower CL	95% Upper CL	p-value	Overall p-value[2]
IL17	Log IL17	0.948	0.910	0.988	0.0110	
CXCL9	Log CXCL9	1.071	1.010	1.134	0.0210	0.0368 (2 df)
	Log CXCL9^2	1.021	1.004	1.039	0.0170	
CXCL10	Log CXCL10	0.941	0.889	0.995	0.0342	0.0054 (2 df)
	Log CXCL10^2	0.964	0.942	0.987	0.0018	
MMP3	Log MMP3	1.336	1.229	1.452	< 0.0001	< 0.0001 (2 df)
	Log MMP3^2	0.916	0.865	0.969	0.0021	
DKK3	Log DKK3	1.411	1.188	1.676	< 0.0001	
IL1RL1	Log IL1RL1	1.206	1.087	1.339	0.0004	
CD163	Log CD163	1.139	0.905	1.435	0.2679	

[1] The functional forms of biomarkers' effects were determined by examining martingale plots

[2] Overall test of biomarker effects

Supplemental Table 5. Penalized regression models of cGVHD with biomarkers and clinical variables

		Hazard Ratio		
**Variable**	**Category**	**Group SCAD, All Variables**	**Adaptive Group Lasso, All Variables**	**Group SCAD, Clinical Variables Only**
LogIL17, BM		1	1	NA
LogIL17, PB		1	1	NA
LogCXCL9, BM		1	1	NA
LogCXCL9, PB		1	1.012	NA
LogCXCL10 BM		1	1.021	NA
LogCXCL10^2, BM		1	0.985	NA
LogCXCL10, PB		1	1	NA
LogCXCL10^2, PB		1	1	NA
LogMMP3, BM		1.352	1.307	NA
LogMMP3, PB		1.013	1.024	NA
LogDKK3, BM		1	0.850	NA
LogDKK3, PB		1.269	1.373	NA
Log IL1RL1, BM		1	1.260	NA
Log IL1RL1, PB		1	1	NA
LogCD163, BM		1	1	NA
LogCD163, PB		1	0.895	NA
Age (years)		1.040	1.027	1.052
Age^2		0.9996	0.9997	0.9995
Graft Source	Bone Marrow	1	1	1
	Peripheral Blood	1.631	1.621	1.553
Primary Disease	AML	1	1	1
	ALL	1	0.922	1
	MDS/MPN/MPS	1	1.074	1
	Lymphoma	1	1.162	1
	Other Malignant	1	1.287	1
	Nonmalignant	1	0.965	1
HLA Matching	8/8	1	1	1
	6-7/8	1	1	1
Conditioning Regimen Intensity	MAC	1	1	1
	RIC/NMA	1	1	1
GVHD Prophylaxis	CNI/MTX	1	1	1
	CNI/MMF	1	0.960	1
	PTCy	1	1.942	1
	Other	1	0.900	1
ATG Use	No	1	1	1
	Yes	1	0.897	1
Donor-Recipient Sex	Not F-M	1	1	1
	F-M	1.403	1.399	1.396
Acute GVHD by Day 90	No	1	1	1
	Yes	1.037	1.086	1.284

Supplemental Table 6. Time-varying Area Under the ROC Curve (AUC(t)) for machine learning models of cGVHD with biomarkers and clinical variables

	AUC(t) Estimate (95% CI)			
**ML Method**	**Day 180**	**Day 270**	**Day 360**	**Day 540**
Group SCAD	0.602 (0.524-0.680)	0.645 (0.581-0.709)	0.666 (0.603-0.728)	0.602 (0.530-0.675)
Adaptive Group Lasso	0.646 (0.574-0.718)	0.654 (0.590-0.717)	0.667 (0.604-0.730)	0.614 (0.540-0.688)
CoxXGBoost	0.626 (0.552-0.700)	0.672 (0.609-0.734)	0.687 (0.625-0.749)	0.650 (0.580-0.719)
Random Comp Risk Forest	0.636 (0.564-0.709)	0.637 (0.573-0.701)	0.684 (0.621-0.746)	0.642 (0.569-0.714)
Oblique Random Surv Forest	0.641 (0.569-0.712)	0.632 (0.567-0.696)	0.656 (0.593-0.720)	0.621 (0.548-0.693)
BART	0.661 (0.589-0.732)	0.667 (0.604-0.731)	0.679 (0.617-0.741)	0.633 (0.561-0.705)
DeepSurv	0.566 (0.494-0.638)	0.578 (0.515-0.641)	0.624 (0.560-0.688)	0.592 (0.521-0.664)
DeepHit	0.571 (0.495-0.648)	0.625 (0.560-0.690)	0.678 (0.614-0.742)	0.641 (0.567-0.715)
Group SCAD: Clin Vars Only	0.585 (0.508-0.661)	0.611 (0.546-0.676)	0.636 (0.571-0.701)	0.581 (0.506-0.656)

Supplemental Table 7. Penalized regression models of moderate/severe cGVHD with biomarkers and clinical variables

		Hazard Ratio		
Variable	Category	Group SCAD, All Variables	Adaptive Group Lasso, All Variables	Group SCAD, Clinical Variables Only
LogIL17, BM		1	1	NA
LogIL17, PB		1	1	NA
LogCXCL9, BM		1	1	NA
LogCXCL9, PB		1	1	NA
LogCXCL10 BM		1	1	NA
LogCXCL10, PB		1	1	NA
LogMMP3, BM		1.712	1.514	NA
LogMMP3, PB		1	1	NA
LogDKK3, BM		1	1	NA
LogDKK3, PB		1	1.316	NA
Log IL1RL1, BM		1	1.273	NA
Log IL1RL1, PB		1	1	NA
LogCD163, BM		1	1	NA
LogCD163, PB		1	1	NA
Age (years)		1	1	1.057
Age^2		1	1	0.9993
Graft Source	Bone Marrow	1	1	1
	Peripheral Blood	1.992	1.966	1.647
Primary Disease	AML	1	1	1
	ALL	1	1.159	1
	MDS/MPN/MPS	1	1.141	1
	Lymphoma	1	0.970	1
	Other Malignant	1	1.527	1
	Nonmalignant	1	0.967	1
HLA Matching	8/8	1	1	1
	6-7/8	1	1	1
Conditioning Regimen Intensity	MAC	1	1	1
	RIC/NMA	1	1	1
GVHD Prophylaxis	CNI/MTX	1	1	1
	CNI/MMF	1	0.796	1
	PTCy	1	2.086	1
	Other	1	0.908	1
ATG Use	No	1	1	1
	Yes	1	1	1
Donor-Recipient Sex	Not F-M	1	1	1
	F-M	1.443	1.391	1.436
Acute GVHD by Day 90	No	1	1	1
	Yes	1	1	1.316

Supplemental Table 8. Time-varying Area Under the ROC Curve (AUC(t)) for machine learning models of moderate/severe cGVHD with biomarkers and clinical variables

	AUC(t) Estimate (95% CI)			
**ML Method**	**Day 180**	**Day 270**	**Day 360**	**Day 540**
Group SCAD	0.572 (0.482-0.661)	0.647 (0.576-0.717)	0.630 (0.563-0.697)	0.593 (0.522-0.664)
Adaptive Group Lasso	0.598 (0.510-0.686)	0.646 (0.575-0.716)	0.620 (0.552-0.687)	0.586 (0.515-0.657)
CoxXGBoost	0.632 (0.547-0.717)	0.670 (0.603-0.738)	0.661 (0.596-0.727)	0.636 (0.567-0.706)
Random Comp Risk Forest	0.618 (0.534-0.703)	0.643 (0.575-0.712)	0.612 (0.545-0.679)	0.587 (0.515-0.658)
Oblique Random Surv Forest	0.625 (0.539-0.710)	0.656 (0.588-0.724)	0.637 (0.571-0.703)	0.597 (0.526-0.668)
BART	0.648 (0.569-0.728)	0.654 (0.586-0.722)	0.630 (0.564-0.696)	0.583 (0.512-0.654)
DeepSurv	0.574 (0.482-0.666)	0.641 (0.571-0.711)	0.646 (0.581-0.712)	0.604 (0.533-0.675)
DeepHit	0.595 (0.508-0.681)	0.654 (0.584-0.724)	0.649 (0.583-0.714)	0.632 (0.562-0.702)
Group SCAD: Clin Vars Only	0.604 (0.514-0.694)	0.632 (0.563-0.702)	0.609 (0.542-0.676)	0.565 (0.493-0.637)

Supplemental Table 9. Penalized regression models of NRM with biomarkers and clinical variables

		Hazard Ratio		
**Variable**	**Category**	**Group SCAD, All Variables**	**Adaptive Group Lasso, All Variables**	**Group SCAD, Clinical Variables Only**
LogIL17, BM		1	1	NA
LogIL17, PB		1	1	NA
LogCXCL9, BM		1	1	NA
LogCXCL9, PB		1	1	NA
LogCXCL10 BM		1	1	NA
LogCXCL10, PB		1	1	NA
LogMMP3, BM		1	1	NA
LogMMP3^2, BM		1	1	NA
LogMMP3, PB		1.297	1.202	NA
LogMMP3^2, PB		0.911	0.939	NA
LogDKK3, BM		1	1	NA
LogDKK3, PB		1	1.014	NA
Log IL1RL1, BM		2.155	2.106	NA
Log IL1RL1, PB		1.794	1.607	NA
LogCD163, BM		2.325	3.012	NA
LogCD163, PB		1	1	NA
Age (years)		1.022	1	1.029
Age^2		1.00002	1	0.999994
Graft Source	Bone Marrow	1	1	1
	Peripheral Blood	1	1.615	1
Primary Disease	AML	1	1	1
	ALL	1	0.817	1
	MDS/MPN/MPS	1	1.048	1
	Lymphoma	1	0.469	1
	Other Malignant	1	0.721	1
	Nonmalignant	1	0.877	1
HLA Matching	8/8	1	1	1
	6-7/8	1.715	1.456	1.889
Conditioning Regimen Intensity	MAC	1	1	1
	RIC/NMA	1	1	1
GVHD Prophylaxis	CNI/MTX	1	1	1
	CNI/MMF	1	1.166	1
	PTCy	1	0.326	1
	Other	1	0.942	1
ATG Use	No	1	1	1
	Yes	1	1	1
Donor-Recipient Sex	Not F-M	1	1	1
	F-M	1	1	1
Acute GVHD by Day 90	No	1	1	1
	Yes	1	1.001	2.068

Supplemental Table 10. Time-varying Area Under the ROC Curve (AUC(t)) for machine learning models of NRM with biomarkers and clinical variables

	**AUC(t) Estimate (95% CI)**			
**ML Method**	**Day 180**	**Day 270**	**Day 360**	**Day 540**
Group SCAD	0.847 (0.742-0.951)	0.817 (0.735-0.900)	0.750 (0.650-0.850)	0.709 (0.613-0.805)
Adaptive Group Lasso	0.878 (0.781-0.975)	0.829 (0.721-0.938)	0.774 (0.672-0.876)	0.738 (0.640-0.836)
CoxXGBoost	0.748 (0.570-0.926)	0.758 (0.631-0.884)	0.744 (0.629-0.858)	0.738 (0.635-0.841)
Random Comp Risk Forest	0.887 (0.808-0.965)	0.809 (0.691-0.927)	0.784 (0.680-0.888)	0.746 (0.653-0.839)
Oblique Random Surv Forest	0.907 (0.846-0.967)	0.808 (0.703-0.913)	0.774 (0.677-0.870)	0.730 (0.642-0.818)
BART	0.817 (0.694-0.941)	0.801 (0.697-0.905)	0.769 (0.669-0.870)	0.743 (0.645-0.840)
DeepSurv	0.855 (0.741-0.969)	0.787 (0.676-0.899)	0.740 (0.641-0.839)	0.676 (0.576-0.776)
DeepHit	0.796 (0.598-0.994)	0.801 (0.668-0.934)	0.777 (0.664-0.889)	0.754 (0.652-0.856)
Group SCAD: Clin Vars Only	0.678 (0.475-0.881)	0.657 (0.523-0.791)	0.650 (0.533-0.766)	0.652 (0.548-0.757)

Supplemental Table 11. BART cGVHD model prediction metrics

Cutpoint[1]	Sensitivity	Specificity	PPV	NPV
0.40	0.765	0.471	0.532	0.593
0.41	0.745	0.493	0.535	0.584
0.42	0.745	0.515	0.546	0.589
0.43	0.717	0.551	0.552	0.588
0.44	0.690	0.596	0.564	0.585
0.45	0.641	0.640	0.577	0.564
0.46	0.579	0.676	0.575	0.544
0.47	0.489	0.721	0.563	0.519
0.48	0.400	0.801	0.588	0.506
0.49	0.386	0.831	0.618	0.506
0.50	0.352	0.838	0.633	0.489

[*] PPV = Positive predictive value, NPV = Negative predictive value

[1] Cutpoint for BART model-predicted cGVHD cumulative incidence at Day 360. Patients with predicted cumulative incidence at or above cutpoint are classified as events for calculation of sensitivity, specificity, PPV, and NPV, while those below the cutpoint are classified as non-events. These metrics were evaluated using the validation cohort.

Supplemental Table 12. BART NRM model prediction metrics

Cutpoint[1]	Sensitivity	Specificity	PPV	NPV
0.01	1.000	0.018	0.075	0.803
0.02	1.000	0.137	0.084	0.780
0.03	0.956	0.291	0.094	0.860
0.04	0.913	0.392	0.103	0.828
0.05	0.870	0.467	0.111	0.819
0.06	0.783	0.546	0.116	0.799
0.07	0.696	0.612	0.117	0.808
0.08	0.696	0.687	0.137	0.812
0.09	0.652	0.731	0.148	0.802
0.10	0.652	0.784	0.174	0.803
0.11	0.565	0.811	0.178	0.784
0.12	0.565	0.868	0.246	0.774
0.13	0.435	0.894	0.235	0.768
0.14	0.435	0.912	0.282	0.763
0.15	0.391	0.925	0.316	0.755

[*] PPV = Positive predictive value, NPV = Negative predictive value

[1] Cutpoint for BART model-predicted NRM cumulative incidence at Day 360. Patients with predicted cumulative incidence at or above cutpoint are classified as events for calculation of sensitivity, specificity, PPV, and NPV, while those below the cutpoint are classified as non-events. These metrics were evaluated using the validation cohort.

Supplemental Table 13. SCAD clinical only model prediction metrics for cGVHD and NRM at the optimal cutpoint

	Cutpoint[1]	Sensitivity	Specificity
**cGVHD**	1.52	58.6%	58.1%
**NRM**	1.63	60.9%	64.8%

[1] Cutpoint is based on the SCAD model-estimated hazard ratio compared to reference group

Supplemental Table 14. Cumulative incidence of cGVHD by BART model-predicted risk groups

	Low Predicted Risk[1] (N = 164)		High Predicted Risk[1] (N = 164)		
**Time Point**	**Estimate**	**95% CI**	**Estimate**	**95% CI**	**p-value[2]**
Day 180	13.0%	(8.7%, 19.3%)	22.9%	(17.2%, 30.3%)	< 0.001
Day 360	32.7%	(26.2%, 40.9%)	58.0%	(50.8%, 66.1%)	
Day 540	39.7%	(32.6%, 48.3%)	62.4%	(55.3%, 70.4%)	

[*] Cumulative incidences were estimated by the Aalen-Johansen estimator applied to the validation cohort.
[1] Risk groups were determined using BART model-predicted cGVHD cumulative incidence at Day 360. Patients with predicted Day 360 cumulative incidence at or above 0.45 are classified as high risk, while those below 0.45 are classified as low risk.
[2] Gray's test

Supplemental Table 15. Cumulative incidence of NRM by BART model-predicted risk groups

	Low Predicted Risk[1] (N = 201)		High Predicted Risk[1] (N = 115)		
**Time Point**	**Estimate**	**95% CI**	**Estimate**	**95% CI**	**p-value[2]**
Day 180	1.5%	(0.5%, 4.6%)	5.2%	(17.2%, 30.3%)	< 0.001
Day 360	3.5%	(1.7%, 7.3%)	13.9%	(50.8%, 66.1%)	
Day 540	4.7%	(2.5%, 8.8%)	20.2%	(55.3%, 70.4%)	

[*] Cumulative incidences were estimated by the Aalen-Johansen estimator applied to the validation cohort.
[1] Risk groups were determined using BART model-predicted NRM cumulative incidence at Day 360. Patients with predicted Day 360 cumulative incidence at or above 0.08 are classified as high risk, while those below 0.08 are classified as low risk.
[2] Gray's test

Supplemental Table 16. Cumulative incidence of cGVHD by SCAD Clinical Variables Only model-predicted risk groups

	Low Predicted Risk[1] (N = 162)		High Predicted Risk[1] (N = 166)		
**Time Point**	**Estimate**	**95% CI**	**Estimate**	**95% CI**	**p-value[2]**
Day 180	15.1%	(10.4%, 21.8%)	20.7%	(15.3%, 27.9%)	0.003
Day 360	38.6%	(31.7%, 47.1%)	51.8%	(44.7%, 60.0%)	
Day 540	43.5%	(36.2%, 52.3%)	58.0%	(50.9%, 66.0%)	

[*] Cumulative incidences were estimated by the Aalen-Johansen estimator applied to the validation cohort.
[1] Risk groups were determined using SCAD clinical variables only model-estimated hazard ratio compared to reference group. Patients with estimated hazard ratio at or above 1.52 are classified as high risk, while those below 1.52 are classified as low risk.
[2] Gray's test

Supplemental Table 17. Cumulative incidence of NRM by SCAD Clinical Variables Only model-predicted risk groups

	Low Predicted Risk[1] (N = 203)		High Predicted Risk[1] (N = 113)		
**Time Point**	**Estimate**	**95% CI**	**Estimate**	**95% CI**	**p-value[2]**
Day 180	1.0%	(0.2%, 3.9%)	6.2%	(3.0%, 12.7%)	< 0.001
Day 360	4.5%	(2.4%, 8.5%)	12.4%	(7.6%, 20.2%)	
Day 540	5.7%	(3.2%, 10.1%)	18.7%	(12.7%, 27.4%)	

[*] Cumulative incidences were estimated by the Aalen-Johansen estimator applied to the validation cohort.
[1] Risk groups were determined using SCAD clinical variables only model-estimated hazard ratio compared to reference group. Patients with estimated hazard ratio at or above 1.63 are classified as high risk, while those below 1.63 are classified as low risk.
[2] Gray's test

Supplemental Table 18. BART prediction model calibration metrics

Outcome	Intercept (95% CI)	Slope (95% CI)
Chronic GVHD at Day 360	0.007 (-0.177, 0.192)	1.080 (0.653, 1.506)
Moderate/severe Chronic GVHD at Day 360	0.040 (-0.103, 0.182)	1.039 (0.602, 1.476)
NRM at Day 360	-0.013 (-0.057, 0.031)	1.157 (0.699, 1.616)

[*] Estimates of intercept and slope obtained from linear regression of pseudovalues of Day 360 event status by BART model-predicted probability using the validation dataset.
